# Educational inequalities in stroke knowledge and symptom recognition following a national Danish stroke campaign: a cross-sectional study

**DOI:** 10.1186/s12889-025-25852-w

**Published:** 2025-12-06

**Authors:** Hannah Andersen, Heidi Shil Eddelien, Malini Sagar, Stine Strandkjaer, Else Foverskov, Christina Kruuse

**Affiliations:** 1 Department of Neurology, Neurovascular Research Unit, Copenhagen University Hospital – Herlev and Gentofte, Herlev, Denmark; 2 Department of Public Health, University of Copenhagen, Copenhagen, Denmark; 3 Department of Clinical Medicine, University of Copenhagen, Copenhagen, Denmark; 4 Department of Brain and Spinal Cord Injury and Neurovascular Research Unit, Neuroscience Centre, Copenhagen University Hospital - Rigshospitalet, Valdemar Hansens vej 23, Glostrup, 2600 Denmark; 5 Danish Resuscitation Council, Herlev, Denmark

**Keywords:** Stroke, Awareness, Stroke signs, Public health campaign, Social inequalities

## Abstract

**Introduction:**

Public stroke campaigns may have informational gaps, which are essential to identify to improve outcomes in stroke awareness and response. We evaluated the impact of a national stroke campaign on knowledge of stroke, and symptom recognition, in relation to educational level.

**Methods:**

Six cross-sectional post-campaign surveys were conducted (2019–2022) using a Danish responder panel (*n* = 2000 first survey, *n* = 1000 each of the following) after the launch of a stroke awareness campaign. Using logistic regression analysis we tested associations between campaign recall and each of the three parameters: knowledge of stroke, symptom recognition, and knowledge of correct call-to-action. Analyses were stratified by educational level.

**Results:**

Of 7001 individuals (56.7% women) 44.8% recalled the campaign during the period, and knowledge of stroke, symptom recognition and correct call-to-action increased over time. Campaign recall was associated with increased knowledge of stroke (OR: 1.57, 95% CI: 1.42–1.74) and symptom recognition (OR: 2.11, 95% CI: 1.89–2.36). The stratified analyses showed that individuals with a master’s degree had higher OR for symptom recognition (OR: 2.59 95% Cl: 1.95–3.44) compared to individuals with vocational education (OR 1.93, 95% Cl: 1.59–2.35).

**Conclusions:**

Initiation of a national stroke campaign was associated with improved knowledge of stroke and symptom recognition. Among individuals with lower educational level the association between campaign recall and recognition of stroke symptoms appeared weaker. The impact of educational levels may be considered in the design of future stroke campaigns. Whether stroke recognition and knowledge of correct call-to-action translate into appropriate responses remains to be fully evaluated.

**Supplementary Information:**

The online version contains supplementary material available at 10.1186/s12889-025-25852-w.

## Introduction

Multimedia campaigns utilizing clear messaging and acronyms like F.A.S.T. (Face, Arm, Speech, Time) have been shown to improve public recognition of stroke symptoms and knowledge of correct call-to-action [1–5]. Improved knowledge may increase the number of individuals who respond correctly to stroke symptoms [1–5]. However, public stroke awareness campaigns often more effectively reach individuals with higher socioeconomic status (SES) [6–8], underscoring the need to target individuals with lower SES who are at elevated risk of stroke [9].

Stroke represents a time-critical condition where delays in identification, correct action, diagnosis, and treatment are associated with poorer clinical outcome [10–14]. Timely access to stroke therapy largely depends on the general population, either by patients or bystanders, identifying stroke symptoms and initiating appropriate call-to-action [15]. Delays in seeking emergency assistance may reflect inadequate symptom recognition or hesitation to contact Emergency Medical Services (EMS) [15–20].

Globally, one in four adults aged 25 and above is expected to experience a stroke during their lifetime [19]. Additionally, more than 101 million individuals are currently living with a previous stroke [19]. While revascularization therapies are effective, they are constrained by narrow therapeutic time windows: 4.5 h with intravenous thrombolysis, and up to six hours for endovascular thrombectomy, respectively [20, 21]. In Denmark approximately 12,000 individuals experience a stroke annually. Although 50% of patients are admitted within 4.5 h of symptom onset, only 23% receive attempted revascularization [22, 23].

Prehospital system delays are longer among individuals with lower SES. The median delay for low-SES patients is 3 h and 47 min (95% CI: 3h30m-4h05m), compared to 3 h and 17 min (95% CI: 3h00m-3h37m) among high-SES patients [24].

Stroke campaigns have been shown to be less effective among individuals with lower educational attainment, indicating a potential social disparity in campaign impact [6–8]. One study found significant increases in symptom recognition among individuals with higher educational level, but not among those with lower education levels [6]. Similarly, individuals with 12 or more years of education were significantly more likely to recognize two or more stroke symptoms following campaign exposure compared to those with less than 12 years of education (OR: 1.33, 95% CI: 1.23–1.44) [7]. Hence, evidence suggest that associations between campaign exposure and improvements in knowledge of stroke symptoms, and correct call-to-action vary across educational groups and settings [6–8].

The aim of this study was to evaluate the impact of the Danish national stroke campaign “Save the Brain” on stroke knowledge, symptom recognition, and knowledge of appropriate call-to-action. Furthermore, we assessed whether these outcomes varied by educational level.

## Materials and methods

### The national stroke campaign

The national stroke awareness campaign “Save the Brain” was launched in 2019 to increase public recognition of stroke symptoms and the importance of prompt emergency response. The campaign utilized an alliterative slogan in Danish: Stræk Snak Smil (Stretch, Speak, Smile). Each word has a corresponding typical stroke symptom: “stretch” referred to sudden weakness or loss of strength in one arm or leg, “speak” indicated sudden difficulty in finding or pronouncing words, and “smile” reflected facial asymmetry.

A campaign video featuring national celebrities depicted the slogan at a rapid rhythmic pace to facilitate memorability and emphasize the urgency of calling EMS. The campaign was initially distributed in 2019 and subsequently disseminated twice annually from 2020 across various platforms, including television, YouTube, Facebook, Instagram, and digital public displays (see Supplementary Material; Supplementary Figure S2). In 2022, the campaign was expanded to include videos that focused on symptom narratives by patients and bystanders. While the campaign targeted the general population, Facebook distribution was technically and economically limited to users aged 65 years and older, a group at elevated stroke risk. Additionally, individuals aged 35–55 were targeted, reflecting their potential role as caregivers to older family members.

### Study design, data sources and study population

**Fig. 1:**
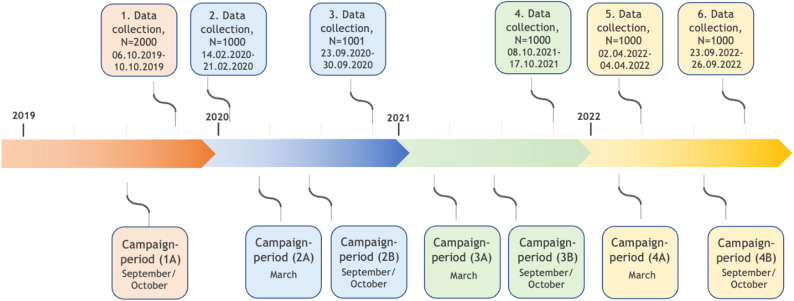
Period for data collection and campaign distribution

Data were obtained from six cross-sectional surveys conducted between 2019 and 2022 among Danish inhabitants aged 18 years and older (Fig. 1). The questionnaires were developed by the Danish Resuscitation Council and administered by an external consultant. The survey instrument was developed specifically for this study. The development process included expert review, as the questionnaire was evaluated for content validity by health professionals. Although a formal pilot test was not conducted, a soft launch was carried out during the first three days of the initial 2019 data collection. During this test the external analysis consultant verified that items were understood correctly and that response patterns were coherent before the survey was released to the full sample. The questionnaire included items on demographics, campaign recall, knowledge of stroke, symptom recognition, and knowledge of correct call-to-action. An English translation of the full questionnaire is provided as Supplementary Material. Individuals who participated in a given survey round were excluded from the immediately subsequent round to prevent duplicate responses. Thus, respondents could only participate in one survey round at a time but might have been eligible for inclusion in later survey rounds. The questionnaires were distributed to randomly selected participants through YouGov’s national online panel in Denmark [25].

### Exposure, outcomes, and covariates

The exposure variable, campaign recall, was categorized based on whether respondents could recall the stroke campaign (“yes”/“no”). Outcome variables were assessed using open-ended questionnaire items requiring free-text responses. An external consultant, in collaboration with the Danish Resuscitation Council, developed a predefined coding scheme and applied it to the responses. Each response could receive up to four codes. The outcome variables included:

- 1: Knowledge of stroke included both ischemic and hemorrhage stroke. Responses coded as “ischemic and hemorrhage stroke” were considered to indicate knowledge of stroke “yes”, while the remaining response categories were grouped together as “no” (see Supplementary Material; Supplementary Table S5-S7).
- 2: Recognition of typical stroke symptoms was defined as: (1) paresis on one side of the body or face, (2) language disturbance, and (3) coordination and balance problems [26, 27]. This categorization was based on the symptoms referenced in the “Save the Brain” campaign. Respondents who identified one or more of these symptoms were categorized as “yes,” while all other responses were grouped as “no”. All correct responses were weighted equally, providing one correct symptom was classified the same as providing multiple correct symptoms.
- 3: Knowledge of correct call-to-action, was defined as calling EMS. The campaign emphasized the need to call 112 immediately in case of stroke. Responses coded as “call EMS/ambulance” were categorized as “yes,” while responses not specifying “call EMS/ambulance were categorized as “no.”

The educational level of respondents was examined as a potential effect modifier. It was defined as the highest completed level of education and categorized as: “master’s degree/Ph.D.,” “bachelor’s degree,” “high school education,” “vocational education,” and “no formal education”. SES was not included because the survey lacked complete and reliable SES indicators, such as household income and employment status. Educational level was therefore used as the primary socioeconomic measure, as it was fully reported and consistently available across survey rounds.

Covariates included sex, age, region of residence, relationship status, prior stroke experience, experience of others having a stroke and time of the survey. Time of survey execution was included as a covariate to address the potential risk of repeated participation by the same individual. Furthermore, to account for secular trends.

### Statistical analysis

The outcome variables were (1) Knowledge of stroke, (2) Symptom recognition and (3) Knowledge of correct call-to-action. Logistic regression models were fitted to examine associations between campaign recall and outcome variables; knowledge of stroke, symptom recognition, and knowledge of correct call-to-action, unadjusted and adjusted for covariates. To examine potential effect modification, analyses were stratified by educational level. Data were weighted by sex, age, and region to reflect the general Danish population [28]. A paired t-test was used to compare a pre-measurement, interpretated before the launch of the campaign, from September 2019 with the post-measurement from September 2022. The statistical significance level was set at < 5%. Results are presented as odds ratio (OR) and corresponding 95% confidence intervals (Cl). Analyses were conducted using R version 4.3.1.

## Results

Overall, 7001 individuals were included in the study. Of these 3137 (44.8%) reported campaign recall (Table 1). Among individuals with campaign recall, 1618 (51.6%) had knowledge of stroke; 2406 (76.7%) recognized stroke symptoms; and 2620 (83.5%) had knowledge of correct call-to-action (Table 1). In total, 3905 (55.8%) did not have knowledge of stroke, 2301 (32.9%) did not recognize stroke symptoms, and 1233 (17.6%) did not have knowledge of correct call-to-action (Table 1).

The mean age was 49.1 years (SD 16.7 years) and 3715 (53.1%) were women (Table 1). The highest completed educational level was distributed as follows: 1145 (16.4%) had a master’s degree, 2679 (38.3%) a bachelor’s degree, 574 (8.2%) a high school education, 2024 (28.9%) a vocational education, and 579 (8.3%) no formal education (Table 1).

**Table 1:** Characteristics of the study population across campaign recall

			Campaign recall			
	Total *N* = 7001		Yes *N* = 3137		No *N* = 3864	
	*N*	%	*N*	%	*N*	%
Sex						
Man	3286	46.9	1357	43.3	1929	49.9
Woman	3715	53.1	1780	56.7	1935	50.1
Age, mean (SD)	49.1	(16.7)	50.0	(16.2)	48.4	(17.0)
Region						
Capital Region	2226	31.8	940	30.0	1286	33.3
Zealand	993	14.2	441	14.1	552	14.3
Southern Denmark	1455	20.8	659	21.0	796	20.6
Central Denmark	1620	23.1	761	24.3	859	22.2
Northern Denmark	707	10.1	336	10.7	371	9.6
Civil status						
Married	3141	44.9	1492	47.6	1649	42.7
In a relationship	1634	23.3	681	21.7	953	24.7
Single	2226	31.8	964	30.7	1262	32.7
Experienced stroke						
No	6671	95.3	2933	93.5	3738	96.7
Yes	330	4.7	204	6.5	126	3.3
Experienced others having a stroke						
No	4225	60.3	1837	58.6	2388	61.8
Yes	2776	39.7	1300	41.4	1476	38.2
Education						
Master/Doc	1145	16.4	437	13.9	708	18.3
Bachelor	2679	38.3	1244	39.7	1435	37.1
Secondary Education	574	8.2	220	7.0	354	9.2
Vocational Education	2024	28.9	940	30.0	1084	28.1
No formal Education	579	8.3	296	9.4	283	7.3
Survey						
October 2019	2000	28.6	935	29.8	1065	27.6
February 2020	1000	14.3	289	9.2	711	18.4
September 2020	1001	14.3	536	17.1	465	12.0
October 2021	1000	14.3	414	13.2	586	15.2
April 2022	1000	14.3	471	15.0	529	14.0
September 2022	1000	14.3	492	15.7	508	13.1
Knowledge of stroke						
No	3905	55,8	1519	48.4	2386	61.7
Yes	3096	44.2	1618	51.6	1478	38.3
Recognition of stroke symptoms						
No	2301	32.9	731	23.3	1570	40.6
Yes	4700	67.1	2406	76.7	2294	59.4
Knowledge of correct call-to-action						
No	1233	17.6	517	16.5	716	18.5
Yes	5768	82.4	2620	83.5	3148	81.5

Characteristics of the study population across campaign recall, whether the respondents recalled the campaign or not

### Change in knowledge over time

Knowledge of stroke increased from 31.7% in 2019 to 52.2% in 2022, representing a significant increase of 20.5% (*p* < 0.001) (Table 2). Furthermore, symptom recognition increased from 40.8% to 72.0%, showing a significant increase of 31.2% (*p* < 0.001). Less improvement was seen for the knowledge of correct call-to-action, which increased from 78.3% to 83.6%, indicating a small but significant increase of 5.3% (*p* < 0.001) (Table 2).

Significant increases in knowledge of stroke and symptom recognition over time were observed for all educational groups (Table 2). Knowledge of the correct actions increased among individuals with a bachelor’s degree from 79.4% to 85.2% (*p* = 0.014) and from 81.0% to 86.9% (*p* = 0.018) among individuals with vocational education (Table 2).

**Table 2:** Change in knowledge of stroke, symptom recognition, and knowledge of correct call-in-action over time, including a pre-measurement

	September 2019 *N* = 2000 *N* (%)	October 2019 *N* = 2000 *N* (%)	February 2020 *N* = 1001 *N* (%)	September 2020 *N* = 1001 *N* (%)	October 2021 *N* = 1000 *N* (%)	April 2022 *N* = 1000 *N* (%)	September 2022 *N* = 1000 *N* (%)	*P*-value
Knowledge of stroke								
Yes	634 (31.7)	877 (43.8)	305 (30.5)	460 (46.0)	438 (43.8)	494 (49.4)	522 (52.2)	< 0,0001
Education								
Master/doc.	107 (34.7)	132 (45.0)	66 (37.9)	92 (50.5)	76 (44.9)	100 (58.4)	88 (56.7)	<0,0001
Bachelor	256 (35.7)	354 (45.9)	134 (34.5)	177 (47.7)	198 (47.0)	183 (52.5)	213 (56.0)	<0,0001
Secondary education	56 (27.4)	62 (40.5)	23 (25.2)	51 (50.0)	33 (44.0)	28 (35.8)	39 (52.0)	0,000
Vocational education	178 (30.1)	255 (42.6)	68 (24.5)	109 (41.1)	102 (38.4)	142 (45.5)	149 (48.5)	<0,0001
No formal education	37 (20.3)	74 (40.0)	14 (20)	31 (38.2)	29 (42.0)	41 (45.0)	33 (39.7)	0,002
Symptom recognition								
Yes	815 (40.8)	938 (65.1)	617 (61.7)	670 (67.0)	658 (65.8)	730 (73.0)	722 (72.2)	< 0,0001
Education								
Master/doc.	139 (45.1)	185 (63.1)	115 (66.0)	126 (69.2)	115 (67.6)	129 (75.4)	109 (70.3)	< 0,0001
Bachelor	319 (44.5)	542 (70.2)	257 (66.2)	268 (72.2)	293 (69.5)	267 (76.7)	301 (79.2)	<0,0001
Secondary education	92 (45.0)	96 (62.7)	51 (56.0)	65 (63.7)	52 (69.3)	53 (67.9)	49 (65.3)	0,002
Vocational education	219 (37.1)	367 (61.3)	156 (56.3)	162 (61.1)	165 (62.2)	221 (70.8)	209 (68.0)	<0,0001
No formal education	46 (25.2)	113 (61.0)	38 (54.2)	49 (60.4)	33 (47.8)	60 (65.9)	54 (65.0)	<0,0001
Knowledge of correct call-to-action								
Yes	1565 (78.3)	1677 (83.9)	789 (78.9)	766 (76.6)	835 (83.5)	865 (86.5)	836 (83.6)	< 0,0001
Education								
Master/doc.	232 (75.3)	243 (82.9)	127 (72.9)	138 (75.8)	135 (79.4)	141 (82.4)	124 (80.0)	0,249
Bachelor	569 (79.4)	650 (84.3)	322 (82.9)	283 (76.2)	356 (84.5)	305 (87.6)	324 (85.2)	0,014
Secondary education	151 (74.0)	129 (84.3)	71 (78.0)	78 (76.2)	63 (84.0)	66 (84.6)	56 (74.6)	0,913
Vocational education	478 (81.0)	506 (84.6)	216 (77.9)	211 (79.6)	227 (85.6)	277 (88.7)	267 (86.9)	0,018
No formal education	135 (74.1)	149 (80.5)	53 (75.7)	56 (69.1)	54 (78.2)	76 (83.5)	65 (78.3)	0,460

*P*-values are from paired t-test from the September 2019 and September 2022

### Associations between campaign recall and knowledge

Individuals with campaign recall demonstrated a higher OR for having knowledge of stroke (OR: 1.57, 95% CI: 1.42–1.74) and symptom recognition (OR: 2.11, 95% CI: 1.89–2.36) compared to individuals without campaign recall (Table 3).

**Table 3:** Association between campaign recall and knowledge of stroke, symptom recognition, and knowledge of correct call-to-action weighted for sex, age, and region, *N* = 7001

	Knowledge of stroke		Symptom recognition		Knowledge of correct call-to-action	
	OR	95% CI	OR	95% CI	OR	95% CI
Unadjusted model						
Campaign recall	1.67	1.51–1.83	2.23	2.01–2.47	1,09	0.96–1.23
Adjusted model						
Campaign recall	1.57	1.42–1.74	2.11	1.89–2.36	1,06	0.93–1.20

All associations are expressed as odds ratios (OR) with their 95% Cl. Adjusted for sex, age, region, marital status, personal history of stroke, experience of others having a stroke, survey, educational level

### Educational differences in associations between campaign recall and knowledge

An educational trend was observed in the associations between campaign recall and symptom recognition, with higher OR for individuals with higher educational level. Individuals with an educational level of master’s degree and campaign recall had a higher OR for recognizing symptoms (OR: 2.59, 95% CI: 1.95–3.44) compared to individuals with a vocational education (OR: 1.93, 95% CI: 1.59–2.35) (Table 4).

**Table 4:** Association between campaign recall and knowledge of stroke, symptom recognition, and knowledge of correct call-to-action stratified on educational levels, weighted for sex, age, and region, *N* = 7001

	Knowledge of stroke		Symptom recognition		Knowledge of correct call-to-action	
	OR	95% CI	OR	95% CI	OR	95% CI
Model 1: Master/doc, *N* = 1145						
Campaign recall	1.39	1.08–1.78	2.59	1.95–3.44	1.27	0.93–1.72
Model 2: Bachelor, *N* = 2679						
Campaign recall	1.78	1.51–2.10	2.17	1.80–2.61	1.08	0.89–1.33
Model 3: Secondary education, *N* = 574						
Campaign recall	1.44	1.01–2.05	2.00	1.37–2.93	0.83	0.54–1.28
Model 4: Vocational education, *N* = 2024						
Campaign recall	1.74	1.44–2.10	1.93	1.59–2.35	0.99	0.75–1.23
Model 5: No formal education *N* = 579						
Campaign recall	0.92	0.64–1.34	2.19	1.51–3.17	1.25	0.81–1.92

All associations are expressed as odds ratios (OR) with their 95% Cl. All models are adjusted for sex, age, region, marital status, personal history of stroke, experience of others having a stroke, survey

## Discussion

This study presents the first evaluation of the Danish national stroke campaign, launched in 2019 and conducted biannually, using the general population as the study population. We found that the national stroke campaign increased knowledge of stroke, recognition of typical stroke symptoms, and knowledge of correct call to action from 2019 to 2022. The increased knowledge was observed across all educational levels. However, individuals with a bachelor’s or master’s degree exhibited larger improvements in knowledge of stroke and symptom recognition compared to other educational groups. Campaign recall was associated with increased knowledge of stroke and correct call-to-action. The impact on symptom recognition appeared to be inversely related to educational level in the stratified analyses, showing less impact with lower education. These findings highlight the need for more research on the differential response to campaigns and suggest that future campaigns should consider strategies to better reach individuals with lower educational level in order to reduce social inequalities in stroke recognition and treatment.

The increases observed from 2019 to 2022 (20.5% in the knowledge of stroke, 31.2% in symptom recognition, and 5.3% in the knowledge of correct call-to-action) demonstrated the accumulated impact of ongoing awareness campaigns (Table 2). Previous research similarly indicates that public stroke campaigns could enhance knowledge of correct call-to-actionalthough such associations may attenuate over time [3].

Other national stroke campaigns, including those applying the F.A.S.T. acronym, have reported improvements in stroke knowledge [1–5]. The results on change in information level suggest that public awareness campaigns can positively influence stroke recognition and knowledge of correct call-to-action. Our findings align with such findings, as campaign recall was associated with increased stroke knowledge and symptom recognition [1–5].

In contrast to previous research, however, we did not observe a significant association between campaign recall and knowledge of correct call-to-action [1–5]. Several factors may contribute to these findings. Our participants may have been exposed to campaign content without consciously recalling it, which could reduce contrast between campaign recall groups. The survey design regarding the order of the questions could have influenced the findings. Respondents were first asked about their knowledge of stroke and then about symptom recognition which may have primed participants to a correct call-to-action response and reduced the contrast between campaign-recall groups. Previous campaign evaluations have addressed unprompted knowledge, or assessed recall before more specific or prompted questions [1, 3, 4]. Additionally, repeated survey participation may have facilitated indirect learning over time prompting knowledge retention.

The differential impact on stroke recognition across educational groups aligned with previous evaluations of stroke awareness campaigns [6]. It was shown that individuals with lower educational level were less likely to recognise stroke symptoms following campaign exposure, and that increases in symptom recognition appeared more pronounced among those with higher educational attainment [6, 7]. Our questionnaire included less commonly associated symptoms with acute stroke, such as headache, nausea, dizziness/loss of balance, or tingling/numbness in the arm/body, which may influence the interpretation of awareness levels.

Previous studies of educational disparities have primarily evaluated indirect impacts, focusing on longitudinal improvements [6–8]. The present approach allowed for a more direct comparison of campaign impact across educational groups, by analysing individual-level association between campaign recall and stroke knowledge, and recognition. Tests of targeted educational interventions further underscores the need for differentiated approaches [29–31]. School-based programs, including FAST Heroes and other structured educational curricula have demonstrated substantial improvements in stroke knowledge among children and families, that may be less effectively reached through mass- media campaigns [29–31]. Also, community-based initiatives have improved stroke preparedness among socioeconomically diverse families, highlighting the value of tailored, context-specific education [30].

National onset-to-arrival data were only available from 2020 onwards, and a simple comparison of data from 2020 to 2023 showed no improvement [32, 33]. This is consistent with a Danish study showing improved symptom knowledge but no reduction in prehospital delay after the first campaign year [34]. Similarly, the PRESTO study indicated that increased awareness did not necessarily shorten time to intervention [35]. A qualitative study investigated pre-hospital behavior among Danish stroke patients and revealed that the stroke response of patients and peers may be driven by symptom severity and emotional response rather than identifying specific symptoms [36]. Notably, the lack of observed behavioral change, despite an increased symptom recognition highlights the need to identify barriers for promptly calling EMS during stroke events. Future studies should identify these barriers using qualitative methods to provide insights into the behavioral changes needed [37].

Social inequalities in knowledge of stroke and symptom recognition have previously been documented [6–8]. It would be highly relevant to investigate how interventions are perceived and understood, and how these translate into action across different educational levels. Applying qualitative approach could ensure representation of all relevant educational groups when identifying specific interventions that address the educational disparities in calling EMS and help identify and mitigate behavioral barriers.

Given the lack of translation from knowledge to actions [3, 34, 35], future campaigns should prioritize correct call-to-action when stroke symptoms occur, regardless of severity [34, 36]. The current study contributes to identify population of interest for future campaigns, including those with lower educational backgrounds. Further, understanding the nuances of demographic groups’ responses to stroke campaigns, the role of ethnicity and need for language and cultural adaptation may be crucial for effectively promoting behavior change.

The following strengths and limitations should be considered when interpreting the findings. A strength of this study was the systematic evaluation of the national stroke campaign in Denmark among the general population. One limitation was the potential sampling bias introduced by use of a self-selected responder panel, where individuals in the YouGov panel participated voluntarily. Efforts were made to mitigate the sampling bias by incorporating weights in the analysis. However, it should be noted that socioeconomic characteristics were not accounted for in the weighting procedure. Moreover, the survey did not include complete or reliable SES indicators such as household income or employment status. As a result, educational level served as the primary socioeconomic measure, which limits the ability to capture the full spectrum of socioeconomic differences and may affect generalizability.

While collecting data over an extended period and including a large sample size (*N* = 7001) was an advantage, stratified analyses by education level would require a larger sample size due to the resulting reduced number of respondents in each subgroup. The smallest group comprised as few as 500 participants, leading to reduced statistical power. As such, careful interpretation and cautious generalization of the findings are warranted.

In conclusion, campaign recall was associated with improved knowledge of stroke and symptom recognition, whereas no significant association was observed for knowledge of correct call-to-action. The association between campaign recall and symptom recognition appeared weaker among individuals with lower educational level, although this trend was not statistically significant. These findings suggest potential educational disparities in campaign impact and highlight the need for further research on how awareness may translate into timely help-seeking behavior, and on how stroke campaigns can more effectively reach groups with lower educational level.

## Supplementary Information

Supplementary Material 1.

Supplementary Material 2.

Supplementary Material 3.

## Data Availability

The data underlying this study are not publicly available due to privacy or ethical restrictions, but they are available from the corresponding author upon reasonable request.
