# Supplementary material for: Educational inequalities in stroke knowledge and symptom recognition following a national Danish stroke campaign: a cross-sectional study
Source: BMC Public Health. 2025 Dec 6;26:148. doi: 10.1186/s12889-025-25852-w (PMC12797781; doi:10.1186/s12889-025-25852-w)
Supplement: Supplementary file 1 — Supplementary Material 1. [file 12889_2025_25852_MOESM1_ESM.docx]

**Outcome: Knowledge of stroke**

| Question: *What is a stroke?* | |
| --- | --- |
| **Coding** | **Category from open text** |
| 0: No | 2: Bleeding or blood clot in the heart |
|  | 3: Bleeding or blood clot in general |
|  | 4: Other |
|  | 5: Don’t know |
| 1: Yes | 1: Bleeding or blood clot in the brain |
| Supplementary table S5: Responses coded as "Bleeding or blood clot in the brain" were considered as those having knowledge about stroke "Yes" (1), while the remaining response categories were grouped as "No" (2-5) and represented a lack of knowledge about stroke. | |

**Outcome: Recognition of stroke symptoms**

| Question: *Which symptoms or warning signs of a stroke (bleeding or blood clot in the brain) do you know, or have you heard of?* | |
| --- | --- |
| **Coding** | **Category from open text** |
| 0: No | 4: Rapid heartbeat |
|  | 5: Chest / arm pain |
|  | 6: Shortness of breath |
|  | 7: Fever |
|  | 8: Vision problems |
|  | 9: Confusion/disorientation/absent-mindedness |
|  | 11: High blood pressure |
|  | 12: Dizziness/loss of balance |
|  | 13: Memory loss |
|  | 16: Fainting/unconsciousness |
|  | 17: Headache |
|  | 18: Nausea/vomiting |
|  | 19: Tingling/numbness in arm/body |
|  | 20: Fatigue |
|  | 21: Breathlessness |
|  | 22: General pain |
|  | 24: Seizure |
|  | 98: Other |
|  | 99: Don’t know |
| 1: Yes | 1: Loss of strength/sensation in arm or leg |
|  | 2: Speech difficulty/slurred/incoherent |
|  | 3: Drooping of mouth corner/facial droop/smiling unevenly |
|  | 10: Paralysis/numbness in general |
|  | 14: General weakness/limited mobility |
|  | 15: Facial paralysis |
|  | 23: One-sided/hemispheric/partial paralysis |
| Supplementary table S6: Respondents who did not recognize one or more stroke symptoms were categorized as "No" (4-9, 11-13, 16-22, 24, 98-99), while those who recognized one or more stroke symptoms were categorized as "Yes" (1-3, 10, 14-15, 23). This change was made to distinguish between responses related to typical stroke symptoms and those that did not. | |

**Outcome: Knowledge of correct call-to-action**

| Question: *Imagine you are in a situation where you witness someone next to you suddenly experiencing symptoms of a stroke (bleeding or blood clot in the brain). What is the first thing you would do?* | |
| --- | --- |
| **Coding** | **Category from open text** |
| 0: No | 2: Lay the person down / sit / place in recovery position |
|  | 3: Check breathing / ensure airway |
|  | 4: Perform chest compressions / first aid / CPR |
|  | 5: Call emergency medical services / doctor on call |
|  | 6: Report symptoms to 112 |
|  | 7: Ask the person to smile / stick out tongue |
|  | 8: Contact relatives / Other |
|  | 9: Request calm / Comfort the person |
|  | 10: Ask for arms over head or stretch / check mobility |
|  | 11: Check speech (Ask person to repeat phrase, answer simple questions) |
|  | 12: Limit secondary injuries / prevent accident |
|  | 13: Get help around / call for assistance |
|  | 14: Establish contact with the person / ask if they are okay / talk to |
|  | 15: Call 114 / 1813 |
|  | 16: Observe/Keep an eye on the person |
|  | 17: Assess symptoms in general |
|  | 18: Check stretch, talk, smile |
|  | 19: Other |
|  | 20: Don’t know |
| 1: Yes | 1: Call 112 / Call for an ambulance |
| Supplementary table S7: Respondents categorized as "Call 112 / ambulance," indicated as "Yes" (1), were considered to have the correct knowledge of call-to-action in case of stroke. The remaining categories were categorized as "No" (2-99), indicating a lack of correct knowledge of call-to-action in case of stroke. | |
