# Supplementary figures and images for: Educational inequalities in stroke knowledge and symptom recognition following a national Danish stroke campaign: a cross-sectional study
Source: BMC Public Health. 2025 Dec 6;26:148. doi: 10.1186/s12889-025-25852-w (PMC12797781; doi:10.1186/s12889-025-25852-w)

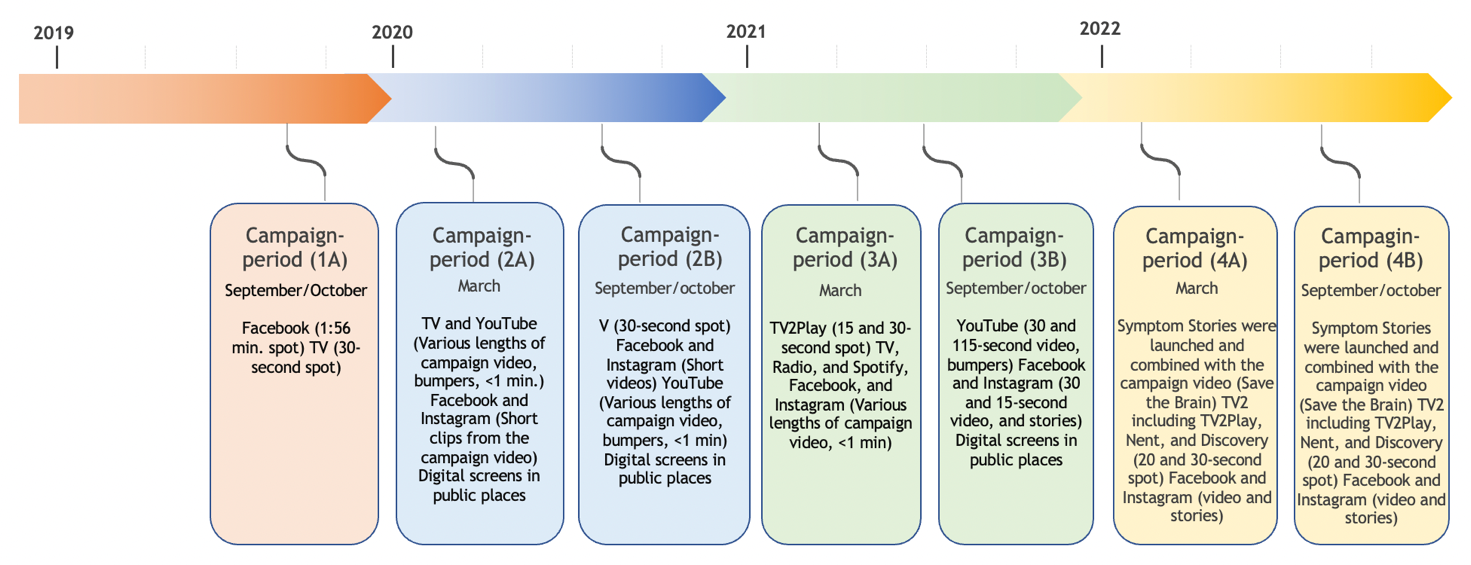


Supplementary Figure S2: Campaign elements, Link to campaign videos: <https://youtu.be/Fdo1bigqtcU>

Supplement: Supplementary file 2 — Supplementary Material 2. [file 12889_2025_25852_MOESM2_ESM.docx]
