# Supplementary material for: Educational inequalities in stroke knowledge and symptom recognition following a national Danish stroke campaign: a cross-sectional study
Source: BMC Public Health. 2025 Dec 6;26:148. doi: 10.1186/s12889-025-25852-w (PMC12797781; doi:10.1186/s12889-025-25852-w)
Supplement: Supplementary file 3 — Supplementary Material 3. [file 12889_2025_25852_MOESM3_ESM.docx]

Danish Council for Resuscitation

Pre- and Post-Measurement Questionnaire 2019

**Questionnaire version v.2.1**

**21 August 2019**

**Purpose:** To assess Danes’ knowledge about bleeding and blood clots in the brain and the concept of “stroke,” including the meaning of “Stretch, Talk, Smile,” as well as to investigate and evaluate the campaign’s impact and effectiveness among all Danes.

**Panel institute:** UserNeeds, YouGov, or Dynata

**Target group:** Persons aged 18+

**Interviews:**

- Pre-measurement: n = 1,000 respondents
- Post-measurement: n = 1,500 respondents

**LOI:** 10 minutes

Text written in italic grey is programming notes.

**Surveys:**

- Questions with red headings are included in both pre- and post-tests.
- Questions with green headings are only included in the post-test.

**Sections**

- Section S: Screening
- Section V: Knowledge
- Section K: Campaign
- Section P: Personal
- Section B: Background information

**Introduction***Type: Information display*

Dear participant,

Thank you very much for taking part in the survey.

We are very grateful for your participation, and your answers will have a significant impact on an upcoming initiative aimed at saving lives.

The interview takes about 10 minutes, and to begin, please click “Next.”

**SEKTION S**S1: Age
*Type: Single*

What is your age?

- Note:__________

S2: Køn
*Type: Single
Randomization: No*

Are you…?

1. Female
2. Male

S3: Region
*Type: Single
Randomization: No*

Which region do you live in?

1. Capital Region of Denmark
2. Region Zealand
3. Region of Southern Denmark
4. Central Denmark Region
5. North Denmark Region

**SEKTION V. Knowledge about stroke**Intro
*Type: Text*

We will now ask you some questions about your knowledge of a disease.

Your answers will be part of a larger study of Danes’ familiarity with the disease, and your responses will therefore have a significant impact on an upcoming initiative aimed at saving lives.

V1: Unaided awareness of stroke

*Type: Open text*

People can suffer a stroke.

What is a stroke?

*Please write in as much detail as possible what you believe a stroke is.*

Note________________

V2: Aided awareness of stroke
*Type: Single
Randomization: Yes*

Which of the following diseases is a stroke?

*Choose only one answer.*

1. Bleeding or blood clot in the brain
2. Bleeding or blood clot in the heart
3. Bleeding or blood clot in the leg
4. Cardiac arrest
5. Cancer
6. High blood pressure
7. Osteoarthritis
8. Diabetes
9. All types of bleeding or blood clot
10. Don’t know (*fixed*)

Info
*Type: Text*

A stroke is bleeding or a blood clot in the brain and is an initial injury to the brain.

V3: Unaided awareness of symptoms
*Type: Open text*

Which symptoms or warning signs of a stroke (bleeding or blood clot in the brain) do you know or have you heard about?

*Please write all the symptoms or warning signs you know or can think of in connection with a stroke.*

Note________________

V4: Aided awareness of symptoms
*Type: Multiple
Randomization: Yes*

Which of the following symptoms or warning signs are the most common when a person suffers a stroke (bleeding or blood clot in the brain)?

*Select all that apply.*

1. Sudden loss of strength in one arm or leg
2. Sudden difficulty finding or pronouncing words
3. One corner of the mouth suddenly droops
4. Severe heart palpitations
5. Severe chest pain
6. Sudden shortness of breath
7. Fever
8. Other, note: :___________ *fixed*
9. Don’t know *exclusive, fixed*

V6. Unaided awareness of Stretch, Talk, Smile
*Type: Text boxes
Randomization: Yes*

What does “Stretch, Talk, Smile” mean in connection with a stroke (bleeding or blood clot in the brain)?

Please write in as much detail as possible what you associate with “Stretch, Talk, Smile.” Stræk ______________

1. Talk ______________
2. Smile ______________

*V7 Randomization*
V7a: Qualified knowledge of stretch
*Type: Single
Randomization: Yes*

By “Stretch” is meant
Select the answer you believe is correct

1. Sudden reduced strength in one arm or leg
2. Sudden neck stiffness
3. Check whether the person can stretch their back
4. Don't know

V7b: Qualified knowledge of talk
*Type: Single
Randomization: Row*

*By ”Talk” it means:*

*Select the answer you believe is correct*

1. Sudden speech difficulty
2. Check if you can get in contact with the person
3. Talk to the person until help arrives
4. Don't know

V7c: Qualified knowledge to smile

*Type: Single
Randomization: Row*

By ”Smile” it means
Select the answer you believe is correct

1. Sudden facial paralysis on one side
2. Sudden toothache in the front teeth
3. Smile at the person to reassure them
4. Don't know

Info
*Type: Text*

The symptoms of a stroke (bleeding or blood clot in the brain) are:

- Sudden reduced or loss of strength in one arm or leg
- Sudden speech difficulty
- Sudden paralysis on one side of the face

V8a: Expected behavior
*Type: Åben besvarelse*

Imagine you are in a situation where a person next to you suddenly experiences the symptoms på et stroke (blødning eller blodprop i hjernen).

What is the first thing you would do?

Note________________

V8b: Expected behavior
*Type: Single
Randomization: Yes*

Imagine you are in a situation where a person next to you suddenly experiences the symptoms suddenly experiences symptoms of a stroke (bleeding or blood clot in the brain).

What is the first thing you would do?
Only choose one answer

1. Call 1-1-2
2. Call your GP
3. Find a defibrillator
4. Start CPR (chest compressions / mouth-to-mouth)
5. Shout for help
6. Check if something is stuck in the throat
7. Wait and see if the symptoms disappear
8. Don't know

V9: Statememts about stroke
*Type: Likert
Randomization: statement*

How much do you agree with the following statements about stroke (bleeding or blood clot in the brain)?

Scale

Strongly agree 2. Somewhat agree 3. Neither agree nor disagree 4. Somewhat disagree 5. Strongly disagree 6. Don’t know

Statement:

I lack knowledge about what to do if I experience, or if someone close to me experiences, a stroke.

SECTION K. Kampagne

K1: Generel campaign knowledge
*Type: Multiple
Randomization: Yes*

Which of the following campaigns can you remember seeing, hearing, or reading about in the past few weeks?
Select all that apply

1. Red Hjernen – stræk, snak, smil
2. Lær de 7 tegn på kræft at kende – og gå til lægen i tide
3. Er du klar til et pust?
4. Hjælp til rygestop
5. Er du lægger?
6. Stop før 5
7. Nederen forældre
8. Spritstiv speeddate
9. Røgfri fremtid
10. Kør bil, når du kører bil
11. Ingen af disse exclusive, fixed
12. Don't know exclusive, fixed

Info
*Type: Text*

We would now like to ask you to watch a film from a campaign. Afterwards, we will ask you a few questions about the campaign film.

Remember to turn on the sound.

[Play the film]

K2: Campaign recall
*Type: Multiple
Randomization: Yes*

Can you remember seeing this campaign film in the past few weeks?

Select all the places where you remember seeing the film.

1. Yes, on TV (e.g., TV2, TV3, etc.)
2. Yes, on Facebook
3. Yes, on YouTube
4. Yes, elsewhere online
5. Yes, in the cinema
6. Yes, but cannot remember where
7. No, do not remember seeing this campaign film in the past few weeks (exclusive, fixed)

K3: Sender recall
*Type: Open text*

Select all that apply

1. Danish Health Authority
2. Danish Cancer Society
3. Hjernesagen (Brain Society)
4. Brain Injury Association
5. Danish Council for Resuscitation
6. TrygFonden
7. Nordea Foundation
8. Danish Heart Foundation
9. Other, please specify

K4: Sender recognition
*Type: Multiple choice*

*Randomization: Yes*

The Danish Council for Resuscitation (DRG) and TrygFonden are the senders of the campaign film.

How clearly do you think it is indicated that the campaign film is from the Danish Council for Resuscitation (DRG) and TrygFonden?

1. Very clear
2. Clear
3. Neither clear nor unclear
4. Unclear
5. Very unclear

K5: Unprompted message understanding
*Type: Open text*

What do you think the Danish Council for Resuscitation (DRG) and TrygFonden wanted to communicate with the campaign film?

Please write as detailed as possible:

Note________________

K6: Prompted message understanding
*Type: Likert*

*Randomization: Statements*

Below are various messages that the Danish Council for Resuscitation (DRG) and TrygFonden aimed to communicate with the campaign film.

Please indicate to what extent you think the campaign film succeeded in conveying each message:

Scale:

1.To a high degree 2. To some degree 3. Neither 4. To a lesser degree 5. Not at all 6. Don’t know

**Statements:**

1. A stroke is an initial brain injury caused by a bleeding or blood clot in the brain.
2. One should check for stroke symptoms using “Stretch, Talk, Smile.”
3. You should immediately call 1-1-2 if there are signs of a stroke.
4. One has a good chance of surviving a stroke and leading a good life if receiving the right treatment in time.
5. You should visit RedHjernen.dk for more information.
6. The three most common signs of a stroke are sudden weakness in one arm or leg, speech difficulties, and facial paralysis.

K7: Liking
*Type: Likert*

*Randomization: No*

Overall, what do you think of the campaign?

1. Very good
2. Good
3. Neither good nor bad
4. Bad
5. Very bad

K7a: Reason for positive liking
*Type: Open text box*

*Logic: If K7 = 1 OR 2*

You mentioned that you liked the campaign. Why did you like the campaign?

Please write as detailed as possible:

Note________________

K7b: Reason for negative liking
*Type: Open text box*

*Logic: If K7 = 4 OR 5*

You mentioned that you disliked the campaign. Why did you dislike the campaign?

Please write as detailed as possible:

Note________________

K8: Interesting, relevant, and credible
*Type: Likert*

*Randomization: Statements*

To what extent do you agree that the campaign film is…

Scale:

Strongly agree 2. Somewhat agree 3. Neither agree nor disagree 4. Somewhat disagree 5. Strongly disagree

Statements:

1. Interesting
2. Relevant
3. Credible

K9: Words describing the campaign
*Type: Multiple choice*

*Randomization: Yes*

Below are words that others have used to describe the campaign. Which words do you think apply to the campaign?

1. Positive
2. Hopeful
3. Fun
4. Strong
5. Emotional
6. Surprising
7. Boring
8. Unpleasant
9. Indifferent
10. Incomprehensible
11. Relevant
12. Friendly
13. Don't know

K10: Word of Mouth (WoM)
*Type: Likert*

To what extent did the campaign film make you want to talk with someone in your family or social circle about stroke (bleeding or blood clot in the brain) and the importance of "Stretch, Talk, Smile"?

1. To a high degree
2. To some degree
3. Neither
4. To a lesser degree
5. Not at all

K11: Information seeking
*Type: Likert*

To what extent did the campaign film make you want to seek more information about stroke (bleeding or blood clot in the brain) and the importance of "Stretch, Talk, Smile"?

1. To a high degree
2. To some degree
3. Neither
4. To a lesser degree
5. Not at all

K12: Sought information
*Type: Multiple*

*Randomization: No*

Have you recently sought more information about stroke (bleeding or blood clot in the brain) and the importance of "Stretch, Talk, Smile" as a result of the campaign?

1. Yes, I visited RedHjernen.dk
2. Yes, I called
3. Yes, I spoke with my own doctor
4. Yes, I searched online
5. Yes, I took a quiz about stroke
6. Other, please specify ______
7. No, I have not sought more information

K13: Other campaign elements
*Type: Multiple*

*Randomization: Yes*

Can you remember seeing other campaign materials about stroke and the importance of "Stretch, Talk, Smile" besides the campaign film you just watched?

1. Quiz on RedHjernen.dk
2. Saw something on a TV program (e.g., Go' Morgen Danmark or Aftenshowet)
3. Saw outdoor posters (e.g., at bus stops or train stations, on buses)
4. Read about it in a leaflet
5. Saw articles about it in newspapers or magazines (print or online)
6. Saw ads in newspapers or magazines (print or online)
7. Heard others talk about it / spoke with others about it
8. Saw something on Facebook
9. Saw something on YouTube
10. Saw it in the cinema
11. Saw it at the doctor's office
12. Other places, please specify ______
13. No, have not seen other materials about stroke or "Stretch, Talk, Smile" (exclusive, fixed)
14. Cannot remember (exclusive, fixed)

SECTION P. Personal

P1: Experienced personally

*Type: Single*

Have you ever had a stroke (bleeding or blood clot in the brain)?

1. Yes
2. No
3. Prefer not to say

P2: Others
*Type: Single*

Has anyone in your family or close circle ever had a stroke (bleeding or blood clot in the brain)?

1. Yes
2. No
3. Prefer not to say

SECTION B. Background
Type: Info

Many thanks for your answers so far. We greatly appreciate your help.

Before we finish, we just need a little background information about you.

B1: Postal code

What is your postal code?

- Note_____

B2: Marital status

What is your marital status?

1. Married
2. Partner (living together)
3. Partner (not living together)
4. Single
5. Widow / widower
6. Prefer not to say / Other

B3: Education

What is your highest completed level of education?

- No education
- Primary school grades 8–10
- General upper secondary education (incl. HF)
- Vocational upper secondary education (incl. HHX and HTX)
- Vocational training (e.g., EUD, craft training, office training, retail training)
- Higher education (up to 2 years – requires prior upper secondary or vocational upper secondary education)
- Higher education (2–4 years)
- Higher education (5 years or longer)

B4: Employment status

*Type: Single
Randomization: No*

What is your primary employment status?

1. Paid employee (full-time, 30 hours or more per week)
2. Paid employee (part-time, under 30 hours per week)
3. Temporary or seasonal employment
4. Self-employed
5. Unemployed – seeking work
6. Unemployed – not seeking work (early retirement, etc.)
7. Retired
8. Homemaker
9. Student / pupil
10. None of the above

B5: Household income

*Type: Single
Randomization: No*

On which of the following levels would you place your household’s total annual income (før skat og andre fradrag)?

1. Up to 99.999 DKK
2. 100.000 – 199.999 DKK
3. 200.000 – 299.999 DKK
4. 300.000 – 399.999 DKK.
5. 400.000 – 499.999 DKK
6. 500.000 – 599.999 DKK
7. 600.000 – 699.999 DKK
8. 700.000 – 799.999 DKK
9. 800.000 – 899.999 DKK
10. 900.000 eller derover
11. Don't know

Info
*Type: Text*

Thank you very much for participating in the survey.

If you have comments on this questionnaire, you are welcome to write them here______
